# The status and high risk factors of severe psychological distress in migraine patients during nCOV-2019 outbreak in Southwest China: a cross-sectional study

**DOI:** 10.1186/s10194-020-01168-5

**Published:** 2020-08-12

**Authors:** Mengmeng Ma, Jinghuan Fang, Changling Li, Jiajia Bao, Yang Zhang, Ning Chen, Jian Guo, Li He

**Affiliations:** Department of Neurology, West China Hospital, Sichuan University, Chengdu, China

**Keywords:** COVID-19, Migraine, Severe psychological distress, Kessler 6-item psychological distress scale

## Abstract

**Background:**

Psychological distress is highly prevalent among migraineurs during public health emergencies. The coronavirus disease 2019 (nCOV-2019) has created mass panic in China due to its highly contagious by contact and aerosols and lack of effective treatment. However, the emotion status of migraineurs stayed unclear during the nCOV-2019 outbreak.

**Objective:**

To understand psychological distress of migraineurs by comparing with common population and identify potential high-risk factors of severe psychological distress among migraine patients.

**Method:**

We enrolled the migraineurs treated at the department of Neurology of West China Hospital and healthy controls with age- and sex-matched to migraineurs. Data on clinicodemographics and psychological distress in the month of February 2020 (during in the nCOV-2019 outbreak in China) were collected. We used the Kessler 6-item (K-6) scale to assess psychological distress. Potential risk factors of severe psychological distress were identified using univariate and multivariate logistic regression.

**Results:**

The 144 migraineurs and 150 controls were included in the study. Migraineurs showed significantly higher K-6 scores than controls (*P* < 0.001). Migraine attack frequency in previous 30 days and time spent paying attention to outbreak showed significant in multivariate logistic regression with respective odds ratios of 2.225 (95%CI 1.361–3.628, *P* = 0.001) and 1.589 (95% 1.117–2.26, *P* = 0.01).

**Conclusion:**

During public health outbreaks, healthcare professionals should focus not only on controlling and reducing migraine attack but also on mental health of migraineurs, especially those with high frequency migraine attack.

## Background

Migraine is a prevalent and disabling neurological disorder that is characterized by recurrent headache, sensitivity to light, nausea, and reduced functioning [1, 2]. Migraine affects 1 out of every 10 individuals among the whole world [3]. In the Global Burden of Disease Study 2017(GBD2017), migraine was ranked 7th based upon years lived with disability (YLDs) and ranked 2nd in the global burden of neurological disorders based on YLDs, which was higher than epilepsy and Alzheimer’s disease [4, 5]. Moreover, migraine was affected by several risk factors, especially emotion factors, such as anxiety and panic [6–8]. Anxiety and panic may increase migraine attack which increase migraineurs’ economic burden [1, 6–8]. Therefore, it is necessary to know the emotion status of migraineurs and study the factors which may affect emotion status of migraineurs.

Since coronavirus disease 2019 (nCOV-2019) was first reported on 08 December 2019 in Wuhan, China [9], it is caused by a novel coronavirus, named COVID-19 or SARS-CoV-2 [10, 11]. nCOV-2019 has created mass panic in China and other countries due to its highly contagious by contact and aerosols and the lack of effective treatment. Globally, more than 3 million confirmed cases with nearly 200,000 deaths were caused by nCOV-2019 on Mar 11, 2020 [12]. Now the outbreak of nCOV-2019 has spread around the whole world and defined as a major public health emergency [13, 14]. Chinese citizens were asked to stay home quarantine during this nCOV-2019 event. Therefore, they can only communicate and get information from media, while almost all the media were focus on nCOV-2019 outbreak. Although scientifically framed information can help people understand and prevent the developing situation of nCOV-2019 [15], excessive attention to media reports about public health emergency can increase risk of psychiatric disorder [16–19]. Anxiety and panic in the public can be created by the flood of disaster-related information, and the mental health of populations can be affected, even threatened by these circumstances, based on World Health Organization assessment [20]. However, the emotion status of migraineurs stayed unclear when facing public health emergency.

While anxiety and panic may increase migraine attack, then serious headache may increase psychiatric disorder in migraineurs [6, 7]. Therefore, we propose our hypothesis that migraineurs would have more trouble controlling their emotion than common population when facing public health emergency. The K-6 scale is a self-rating scale created by Kessler and his colleagues for preliminary screening of whether respondents have serious psychological diseases. The Mandarin version was validated in the World Mental Health Survey 25 and shows good reliability and validity [21].

The present study compared the severity of psychological distress between migraineurs and common population during the nCOV-2019 outbreak in southwest China by using Kessler 6-item psychological distress scale and a custom-designed questionnaire. It also explored risk factors for severe psychological distress among migraineurs, which showed the frequency of migraine attacks and attention paid to media coverage of the nCOV-2019 outbreak might cause severe psychological distress in migraineurs.

## Method

This is a cross-sectional case control study. Respondents were asked the questionnaire information about the month of February 2020, most into the COVID-19 outbreak in China.

### Study participants

All the patients were recruited from the Department of Neurology of West China Hospital. All the participants were 18–60 years of age and have been diagnosed with migraine without aura at least 1 year before, the treatment of migraine was unchanged and did not take any other drugs regularly for 3 months at least. Moreover they had to be followed up monthly by a neurology specialist. Patients were excluded if they were confirmed COVID-19; or they were diagnosed with other neurological or psychiatric disorders; or had a history of alcohol or drug abuse, or uncontrolled psychosis; or they were unable to read or understand the questionnaires. The diagnosis of migraine without aura was made according to the International Classification of Headache Disorders, 3rd edition (ICHD-3).

In parallel, we invited a control group of healthy visitors of patients who were unrelated to those patients. Healthy controls were age- and sex-matched to migraine patients. The healthy controls had no history of migraine or other types of headache, psychiatric disorders, and alcohol or drug abuse and did not take any other drugs regularly for 3 months at least also.

The study protocol was approved by the Local Ethics Committee of West China Hospital, Sichuan University. The participants gave informed consent.

### Survey instruments

Enrolled participants were sent on-line questionnaires via WeChat and were asked to fill them out.

#### Questionnaire on demographics, clinical data, epidemiological contact information

The questionnaire for this study included the demographics information about sex, age, marriage status, education level; clinical data about migraine information (attack frequency), medical history (including mental illness history, and treatment history) from electronic clinical records in the West China Hospital outpatient department; and epidemiological contact information about their level of concern over the COVID-19 outbreak (on a 4-point scale from “relatively unconcerned or very unconcerned” to “very concerned”), and the time they spent each day receiving information about the outbreak.

#### Kessler 6-item psychological distress scale (K-6)

The K-6 scale is a self-rating scale created by Kessler and his colleagues for preliminary screening of whether respondents have serious psychological diseases. It has been validated to assess non-specific psychological distress during the past 30 days [22], and it is often used in large-scale investigations [23] . The Mandarin version was validated in the World Mental Health Survey 25 and shows good reliability and validity [21]. The scale includes 6 psychological symptoms: “nervous”, “hopeless”, “restless or fidgety”, “so depressed that nothing could cheer you up”, “Everything is an effort” and “worthless” [22]. The Link5 score is adopted, “0” means no time, “1” means occasionally, “2” part of time, “3” means most of time, and “4” means all the time, with a total score of 0 ~ 24 [24]. Severe psychological distress was defined as a total score > 12 [21].

### Statistical analysis

Statistical analysis was performed using SPSS 22.0 (IBM, Chicago, IL, USA). The values are reported as absolute numbers with percentages for categorical variables and as means with SDs for continuous variables. Χ ^2^ tests and t-tests were performed to compare categorical variables between groups, continuous variables between two groups respectively. K-6 scores showed a skewed distribution, so the rank sum test was used to compare the two groups. Univariate logistic regression was used to determine the association between clinicodemographic factors and severe psychological distress. Multivariate logistic regression was used to explore factors independently associated with severe psychological distress in patients with migraine. The multivariate model contained variables that were associated with *p* < 0.1 in univariate analysis.

The level of statistical significance was defined as combined *P* < 0.05.

## Results

Of the 160 patients with migraine participated in the survey, 7 were excluded because they had mental disease before, 8 because they had alcohol or drug abuse and 1 because the patients were confirmed case of COVID-19. In the end, 144 migraine patients (90%) were enrolled. Then we invited 150 healthy controls with age- and sex-matched to migraine patients.

### Demographic and clinical characteristics

Subject demographics and clinical characteristics were shown in Table 1. Patients and healthy controls showed no significant differences in sex, age, marriage status, education level, history of epidemiological contact, suspected COVID-19 cases, COVID − 19 cases in respondent’s residential community and feeling difficulty to control emotion during the nCOV-2019 outbreak. Compared to healthy controls, Patients reported significantly lower family income (*P* < 0.001). Besides, migraine patients reported significantly greater concern about the outbreak (*P* < 0.001) and more time paying attention to outbreak (*P* < 0.001) and higher frequency of dreaming about nCOV-2019 (*P* = 0.019) than healthy controls.

**Table 1:** Clinicodemographic characteristics of participants

characteristic	Migraine patients (*n* = 144)	Healthy controls (*n* = 150)	*P*
Male	32 (22%)	39 (26%)	0.449
Age, year	36.6 ± 8.4	36.8 ± 9.5	0.88
Marriage	98 (68%)	119 (79%)	0.088
Education level, year			
≤ 12	59 (41%)	50 (33%)	0.175
> 12	85 (59%)	100 (67%)	
Family monthly income ^a^, RMB			
0–4999	31 (22%)	37 (25%)	< 0.001
5000–9999	41 (29%)	51 (34%)	
10,000–14,999	52 (36%)	34 (23%)	
15,000–19,999	12 (8%)	11 (7%)	
> 20,000	8 (6%)	16 (11%)	
Duration of migraine, y	7.41 ± 5.72		
Migraine attack frequency in previous 30 days	3.58 ± 3.28		
History of epidemiological contact	4 (3%)	4 (3%)	0.953
Suspected COVID-19 cases	21	12	0.074
COVID-19 cases in respondent’s residential community	23 (26%)	15 (10%)	0.127
Level of concern about 2019-nCoV outbreak			
Very concerned	95 (66%)	67 (45%)	< 0.001
Concerned	32 (22%)	68 (45%)	
Average	17 (12%)	12 (8%)	
Relatively unconcerned or very unconcerned	0 (0)	3 (2%)	
Time spent paying attention to outbreak, hours per day	2.3 ± 1.5	1.5 ± 1.0	< 0.001
Feeling difficulty to control emotion during the 2019-nCoV outbreak	18 (13%)	11 (7%)	0.137
Frequency of dreaming about 2019-nCoV			
most of time	10 (7%)	5 (3%)	0.019
sometimes	28 (19%)	15 (10%)	
none	106 (74%)	130 (87%)	

Values are n (%), mean ± SD, or median (maximum, minimum)

^a^1 RMB = 0.143 USD in 29 Feb 2020

### K-6 scores

The K-6 scores were shown in Table 2. Compared with healthy controls, patients with migraine had significantly higher K-6 scores than healthy controls (*P* < 0.001), including for the items about feeling “nervous”, “hopeless”, “restless or fidgety”, “so depressed that nothing could cheer you up”, and “everything is an effort”. In contrast, the two groups scored had no difference on the item about “feeling worthless”. The proportion of patients with severe psychological distress, whose scores were more than 12 (17%), was significantly greater than the controls group (8%) (*P* = 0.016).

**Table 2:** Scores of participants on the Kessler 6-item psychological distress scale

K6 item	Migraine patients	Healthy controls	*P*
Nervous	1 (4,0)	0 (4,0)	< 0.001
Hopeless	1 (3,0)	0 (4,0)	< 0.001
Restless or fidgety	1 (4,0)	0 (3,0)	< 0.001
So depressed that nothing could cheer you up	1 (4,0)	0 (3,0)	< 0.001
Everything is an effort	1 (2,0)	0 (2,0)	< 0.001
Worthless	0 (1,0)	0 (2,0)	0.598
Total score	5 (18,0)	0 (16,0)	< 0.001
Severe psychological distress (n,%)	25 (17%)	12 (8%)	0.016

Values are median (maximum, minimum) or n (%)

### Assessment of the clinicodemographic factors associated with severe psychological distress

The results of univariate and multivariate logistic regression were separately shown in Table 3 and Table 4. Univariate analyses showed that the age (*P* = 0.033), migraine attack frequency in previous 30 days (*P* = 0.005), history of epidemiological contact (*P* = 0.018), suspected COVID-19 cases (*P* < 0.001), COVID-19 cases in respondent’s residential community (*P* = 0.004), time spent paying attention to outbreak (*P* < 0.001) and frequency of dreaming about nCOV-2019 (*P* = 0.044). Only migraine attack frequency in previous 30 days and time spent paying attention to outbreak showed significant in multivariate logistic regression with respective odds ratios of 2.23 (95%CI 1.36–3.63, *P* = 0.001) and 1.59 (95% 1.12–2.26, *P* = 0.01).

**Table 3:** Exploration of factors associated with severe psychological distress (K6 score > 12) among migraine patients

Factor	Odds ratio (95%CI)	*P*
Sex	1.62 (0.51, 5.10)	0.414
Age	0.94 (0.88, 1.00)	0.033
Marriage	0.87 (0.40, 1.88)	0.718
Education level	1.29 (0.53, 3.15)	0.794
Family monthly income	1.23 (0.77, 1.99)	0.386
Duration of migraine	1.00 (0.93, 1.08)	0.946
Migraine attack frequency in previous 30 days	7.67 (1.83, 32.12)	0.005
History of epidemiological contact	16.09 (1.6, 161.86)	0.018
Suspected COVID-19 cases	11.24 (4, 32.26)	< 0.001
COVID-19 cases in respondent’s residential community	4.22 (1.57, 11.36)	0.004
Level of concern about 2019-nCoV outbreak	1.16 (0.61, 2.23)	0.654
Time spent paying attention to outbreak, hours per day	2.99 (1.94, 4.63)	< 0.001
Frequency of dreaming about 2019-nCoV	1.91 (1.02, 3.59)	0.044

Reference conditions for OR calculations: female, married, ≤12 years of education, had history of epidemiological contact, had suspected COVID-19, had COVID-19 cases in respondent’s residential community

**Table 4:** Multivariate logistic regression to identify independent predictors of severe psychological distress (K6 score ≥ 12)

Factor	OR	95% CI	*P*
Age	1.04	0.88, 1.06	0.428
Migraine attack frequency in previous 30 days	1.59	1.12, 2.26	0.01
History of epidemiological contact	17.31	0.32, 94.66	0.163
Suspected COVID-19 cases	3.56	0.77, 16.39	0.104
COVID-19 cases in respondent’s residential community	1.58	0.33, 7.59	0.569
Time spent paying attention to outbreak, hours per day	2.23	1.36, 3.63	0.001
Frequency of dreaming about 2019-nCoV	1.38	0.26, 2.03	0.542

Reference conditions for OR calculations: had history of epidemiological contact, had suspected COVID-19, had COVID-19 cases in respondent’s residential community

## Discussion

Migraineurs were more likely to have severe psychological distress than common population. After further explore risk factors related to severe psychological distress in migraineurs during nCOV-2019 outbreak, we found the frequency of migraine attacks and attention paid to media coverage of the nCOV-2019 outbreak is the 2 high risk factors for psychological distress in migraineurs.

### Compare to common population, migraineurs were more likely to have severe psychological distress during nCOV-2019 outbreak

On one hand, Psychological distress among migraineurs has significantly higher level of it than controls. The highest score in Kessler 6-item psychological distress scale of migraineurs is 18, while it is 16 in common population. And the total score of Kessler 6-item psychological distress scale of migraineurs group were also higher than it of common population. On the other hand, there were 17% of migraineurs group and 8% of control group showed severe psychological distress. The percentage of severe psychological distress of both groups are significantly higher than it of the general population (3.97%) in China before nCOV-2019 outbreak [21]. Our results matched the fact that public health emergency can increase risk of psychiatric disorder [16, 18, 25]. Moreover, the percentage of severe psychological distress of common population was also higher than the percentage of post-traumatic stress symptoms in China hardest-hit areas a month after the COVID-19 outbreak (7%) [23]. This may cause by time gap. Liu [26] surveyed at about January, while we surveyed at February 1st. Compare to January, much more suspect and confirm cases of COVID-19 showed in February and Chinese citizens stayed home to for longer time, more media information flooded in [25]. Studies also showed that longer quarantine duration would cause psychological distress [16, 27]. There is an increasing evidence that during public health emergency psychological distress together with alexithymia may be considered risk factors for negative outcomes (e.g., suicide), even simply increasing the risk of development of depressive symptoms [28, 29]. In addition, the unique sensory processing patterns of individuals with major affective disorders and their relationship with psychiatric symptomatology have been clearly reported. Hyposensitivity or hypersensitivity may be “trait” markers of individuals with major affective disorders [30]. Taken together, it indicated that migraineurs may have more trouble controlling their emotions than common population.

### Factors related to severe psychological distress in migraineurs during nCOV-2019 outbreak

The average hours spent daily on media coverage of the nCOV-2019 outbreak in migraineurs are 2.3 h compare to 1.5 h in common population. We found the amount of time spent daily on media coverage of the nCOV-2019 outbreak were associated with severe psychological distress among migraineurs during the nCOV-2019 outbreak. This result matched the fact that higher social media exposure were associated with/increased the risk of psychological distress [16, 31, 32].

We also found that migraine attack frequency in previous 30 days was associated with severe psychological distress among migraineurs during the nCOV-2019 outbreak. It is well known that migraine attack caused migraineurs serious headache, which may cause increase anxiety emotion, such as fear of pain or another attack [7]. And facing nCOV-2019 outbreak was anxiety and panic emotion trigger [16, 25]. Combine with these two factors, this result indicated that during public health emergency, migraineurs should take measures or at least pay more attention to control or reduce migraine attack and should focus on only the necessary media information about the outbreak in order to avoid severe psychological distress.

In addition, migraineurs who suffered from severe psychological distress should consult psychiatrist as soon as possible. It is a challenge for migraineurs to achieve treatment in time during this nCOV-2019 outbreak. Studies have recommended telepsychiatry, including online clinic, social media and the internet, to provide clinical resources and group emotional support for migraineurs [29].

### Limitations of the study

First, we only collected data at south-western China, while currently the epidemic was widespread. However, potential relationships of psychological distress with distance between the epicenter of the epidemic should be taken considered, because such a relationship has been shown for earthquake survivors [33]. Second, psychological distress and time spent daily paying attention to the outbreak were based on self-report, which may increase risk of bias. Third, the details of media were not collected. The different type of media might affect the amount of information that responders got [32]. But this would not change out results. At last, we only include 144 migraineurs and 150 controls. Therefore, our results should be verified and extended in larger patient samples.

## Conclusions

During public health outbreaks, healthcare professionals should focus not only on controlling migraine attack but also on mental health of migraineurs, especially those with high frequency migraine attack. They should advise migraineurs to avoid paying too much attention on media coverage of the outbreak.

## Data Availability

Not applicable. The datasets used and analyzed in the current study are not publicly available. Anonymous data is available from the corresponding author upon reasonable request.

## Abbreviations

GBD2017: Global Burden of Disease Study 2017
YLDs: Years lived with disability
nCoV-2019: Coronavirus disease 2019
K-6: Kessler 6-item
ICHD-3: The International Classification of Headache Disorders, 3rd edition
